# Breakthrough Covid‐19 infections in vaccinated recipients of allogeneic stem cell transplantation

**DOI:** 10.1002/jha2.512

**Published:** 2022-07-14

**Authors:** Slim Fourati, Christine Robin, Christophe Rodriguez, Mathieu Leclerc, Florence Beckerich, Jean‐Michel Pawlotsky, Rabah Redjoul, Sébastien Maury

**Affiliations:** ^1^ Virology Department Assistance Publique‐Hôpitaux de Paris (AP‐HP) Henri Mondor Hospital Créteil France; ^2^ INSERM U955 Paris Est Créteil University UPEC Créteil France; ^3^ Hematology Department AP‐HP Fédération Hospitalo‐Universitaire TRUE InnovaTive theRapy for immUne disordErs Henri Mondor Hospital Créteil France

1

Dear Editor,

Allogeneic hematopoietic stem cell transplant (HSCT) recipients are at high risk of developing severe and/or lethal forms of Coronavirus disease 19 (Covid‐19), the entity described 3 years ago and related to the emergence in China of a novel coronavirus referred to as severe acute respiratory syndrome coronavirus 2 (SARS‐CoV‐2). While hope has arisen a year ago from the promising results of messenger RNA (mRNA) vaccines studies, showing high protection rates and a favorable safety profile, some concerns remain regarding the efficacy of vaccination in HSCT recipients, as humoral response might be altered in this setting because of concomitant immunosuppressive medications, and delay or alteration in B‐cell reconstitution. We and others previously identified that patients in the 3–12 months post‐HSCT developed significantly lower antibody titers after vaccination compared with patients in the > 12 months post‐HSCT group and healthy controls [1, 2], making the first year after HSCT a period of critical susceptibility to breakthrough Covid‐19 infections.

In relation with only progressive immune reconstitution over time as well as immunosuppressive medications needed over this early post‐transplant period, vaccination of recipients never starts before 3 to 6 months after HSCT. Hence, it is usually recommended to cover this critical period with additional prophylactic measures. The Food and Drug Administration has issued an Emergency Use Authorization (EUA) for the investigational long‐acting monoclonal antibodies (mAbs) tixagevimab and cilgavimab (Evusheld‐AstraZeneca) for pre‐exposure prophylaxis of COVID‐19 in persons ≥12 years old who have moderate or severe immune compromise. The French commission for vaccination against Covid‐19 has been recently recommending the use of Evusheld in the particular setting of HSCT patients within the early period following transplantation and for patients with very low humoral response despite vaccination. Figure 1 summarizes prophylactic measures against breakthrough Covid‐19 infections in allogeneic HSCT recipients. Recent evidence of enhanced humoral response to early post‐transplant vaccination, when the donor has been vaccinated before donation, may further improve the overall protection of HSCT recipients, most donors becoming vaccinated over time [3].

**FIGURE 1:**
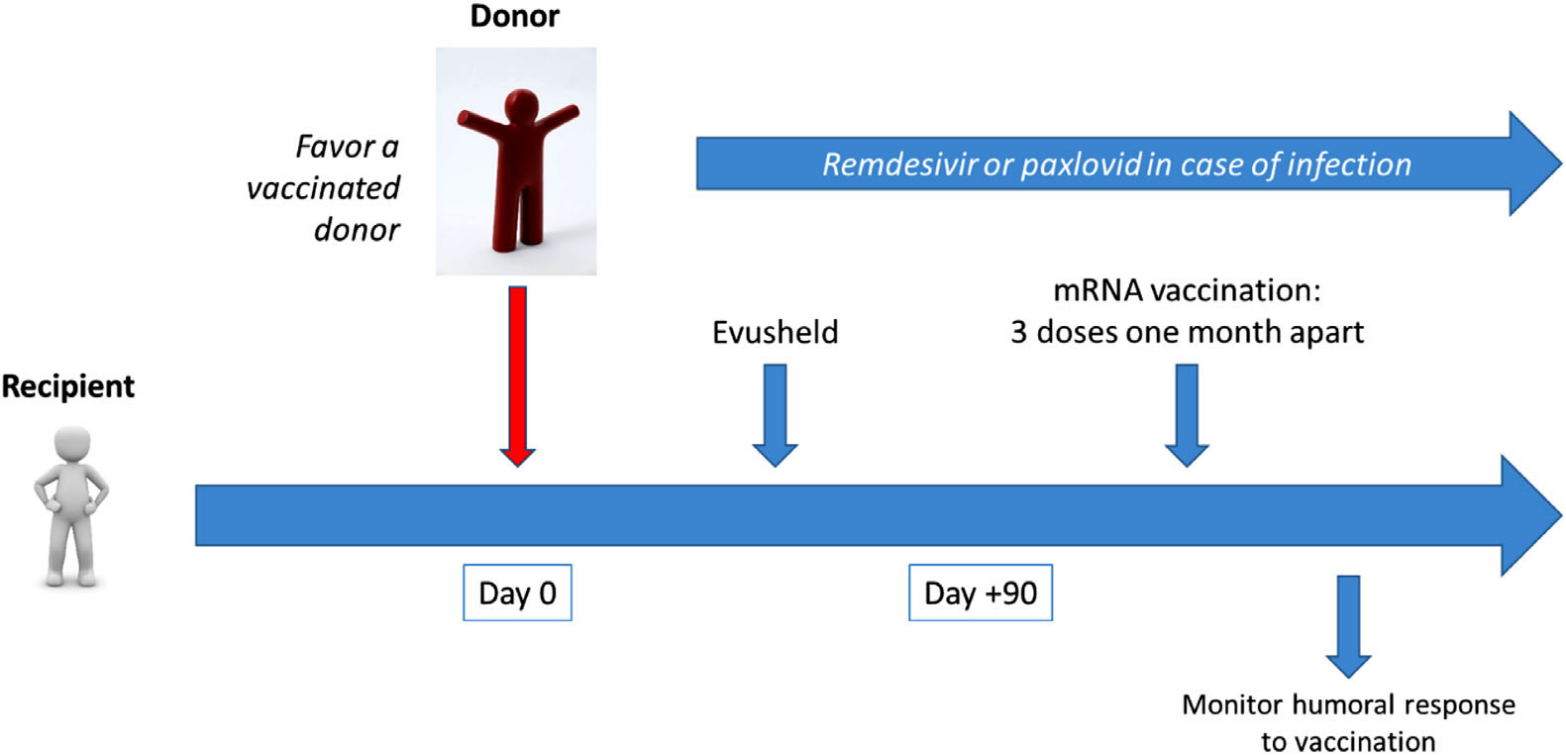
Prophylactic measures against breakthrough Covid‐19 infections in allogeneic HSCT recipients. mRNA vaccination may be typically initiated after a delay of 3 months following HSCT, the preceding period being covered by prophylactic Evusheld infusions. In case the patient has to receive highly immune suppressive drugs in the early months after HSCT (typically rituximab or high dose steroids), vaccination may be delayed in order to favor humoral response together with possible additional Evusheld infusions. In case of infection despite prophylactic measures, curative treatment using remdesivir or paxlovid may be considered

Of importance, prophylactic efficacy of these measures is challenged by emerging immune‐evasive SARS CoV‐2 variants, notably since the SARS‐CoV‐2 Omicron variant has become dominant worldwide. This lineage is characterized by the presence of more than 30 mutations in spike—located mostly in the N‐terminal domain and the receptor‐binding domain—that have been shown to enhance viral attachment to the cellular receptor ACE‐2 and enable antibody evasion. The variant comprises two main dominant sub‐lineages termed BA.1 and BA.2. Sublineage BA.1 can be further classified into several sublineages, the most dominant being BA.1.1 (harboring an R346K substitution as compared to BA.1), which has supplanted the majority of BA.1 variants in Europe. While all Omicron sublineages escape a majority of authorized mAbs in vitro, the efficacy of Evusheld has been suggested to be somehow conserved with varying levels of activity depending on the Omicron sublineage [4, 5]. In clinical practice, the efficacy of Evusheld in preventing Omicron infection in immunocompromised patients remains unclear.

Among the first three HSCT recipients receiving Evusheld as a pre‐exposure prophylaxis in our center, we observed two cases of breakthrough Omicron infection. Both presented at our department on January 2022 with fever, myalgia and upper respiratory symptoms. They previously received Evusheld prophylaxis (one dose of 300 mg) based on their low humoral response after three post‐transplant BNT162b2 mRNA vaccine doses (Table 1). Full‐length SARS‐CoV‐2 genomes were sequenced from nasopharyngeal swabs at diagnosis using the COVIDSeq Test (Illumina, San Diego, California) and DRAGEN server, evidencing BA.1.1 sublineage harboring R346K substitution in both cases. These cases illustrate that administration of Evusheld in immunocompromised HSCT recipients does not enable to neutralize BA.1.1 sublineage, in agreement with in vitro studies showing poor seroneutralization activity of Evusheld against BA.1.1, mainly due to R346K substitution [4]. Importantly, our two HSCT recipients had mild disease suggesting that administered mAbs could help prevent severe forms of the disease. A recent study also reported 4 breakthrough Omicron infections among 29 Evusheld‐treated immunocompromised patients [5]. However, viral genomes were not sequenced in the latter study, except in one patient infected with sublineage BA.1. We did not perform in vitro seroneutralization assays in these two patients. However, the anti‐S RBD concentrations provided by the assay (Abbott) have been shown to strongly correlate with the level of neutralizing antibodies. When validating the assay, we further observed strong correlations between Abbott anti S‐RBD concentrations and in vitro neutralizing activity against several variants in allogeneic HSCT recipients.

**TABLE 1:** HSCT recipients developing SARS‐CoV‐2 Omicron BA.1.1 infection despite prophylaxis by Evusheld™

Gender	Disease	Age (years)	Time elapsed from HSCT (days)	Time elapsed from Evusheld™ injection (days)	GVHD requiring systemic immune suppression	IgG(S‐RBD) titer after third vaccine dose post‐HSCT (AU/ml)	IgG(S‐RBD) titer at time of infection (AU/ml)	Hospital admission	Specific treatment of infection
F	AML	59	251	9	Yes	97	8433	No	None
M	AML	69	217	11	Yes	923	24776	Yes	Remsedivir

Sensitivity of the next dominant variants to monoclonal antibodies is of highest importance. Hopefully, in contrast to BA.1, Evusheld seems to act efficiently, at least in vitro, against BA.2. As BA.2 is rapidly replacing BA.1 (and BA.1.1) worldwide, the activity of Evusheld should be gained against this sublineage. However, the risk that further escape mutations will arise is high and the capacity of available mAbs (including Evusheld, sotrovimab, and new mAbs) to neutralize emerging variants should be continuously evaluated in vitro as well as in vivo in recipients of allogeneic HSCT. Additionally, specific determinants of waning immunity in this high‐risk patient population such as low peak titers of anti‐spike glycoprotein‐specific IgG after primary vaccination [6] may be used in order to anticipate additional delayed vaccine doses and/or reinfusion of neutralizing monoclonal antibodies.

## FUNDING INFORMATION

The authors received no specific funding for this work.

## CONFLICT OF INTEREST

The authors declare that there is no conflict of interest that could be perceived as prejudicing the impartiality of the research reported.

## Data Availability

Viral sequences in the two patients have been submitted to Gisaid database.
